# The Role of the Extracellular Matrix in the Pathogenesis and Treatment of Pulmonary Emphysema

**DOI:** 10.3390/ijms251910613

**Published:** 2024-10-02

**Authors:** Jerome Cantor

**Affiliations:** School of Pharmacy and Allied Health Sciences, St John’s University, Queens, NY 11439, USA; jocantor1@gmail.com

**Keywords:** pulmonary emphysema, extracellular matrix, elastin, collagen, hyaluronan

## Abstract

Pulmonary emphysema involves progressive destruction of alveolar walls, leading to enlarged air spaces and impaired gas exchange. While the precise mechanisms responsible for these changes remain unclear, there is growing evidence that the extracellular matrix plays a critical role in the process. An essential feature of pulmonary emphysema is damage to the elastic fiber network surrounding the airspaces, which stores the energy needed to expel air from the lungs. The degradation of these fibers disrupts the mechanical forces involved in respiration, resulting in distension and rupture of alveolar walls. While the initial repair process mainly consists of elastin degradation and resynthesis, continued alveolar wall injury may be associated with increased collagen deposition, resulting in a mixed pattern of emphysema and interstitial fibrosis. Due to the critical role of elastic fiber injury in pulmonary emphysema, preventing damage to this matrix component has emerged as a potential therapeutic strategy. One treatment approach involves the intratracheal administration of hyaluronan, a polysaccharide that prevents elastin breakdown by binding to lung elastic fibers. In clinical trials, inhalation of aerosolized HA decreased elastic fiber injury, as measured by the release of the elastin-specific cross-linking amino acids, desmosine, and isodesmosine. By protecting elastic fibers from enzymatic and oxidative damage, aerosolized HA could alter the natural history of pulmonary emphysema, thereby reducing the risk of respiratory failure.

## 1. Introduction

Pulmonary emphysema is characterized by low-grade inflammation involving the release of enzymes that degrade the extracellular matrix (ECM), a complex network of proteins, glycoproteins, and proteoglycans that surrounds the cellular components of the lung and provides the structural support necessary for efficient gas exchange [1,2,3,4,5]. Damage to the interstitial ECM can impair lung mechanical recoil and increase the air pressure within alveoli, causing distention and rupture of their walls, with a resultant loss of gas exchange. Initially, the damage to the ECM may only involve isolated foci within the lung parenchyma. However, as the injury progresses, increasingly uneven transmission of forces exerts greater strain on alveolar walls, producing widespread airspace enlargement.

A prominent feature of pulmonary emphysema is an imbalance between ECM synthesis and degradation, involving increased expression of ECM-degrading enzymes, such as matrix metalloproteinases (MMPs) [6,7]. These enzymes break down various ECM components, including elastic fibers and collagen, leading to alveolar wall rupture [8,9]. The release of the fragmented matrix components perpetuates this process by promoting the recruitment and activation of inflammatory cells [10].

The recognition that enzymatic injury to the ECM is an important component of the disease has led to the development of therapeutic interventions that prevent the destruction and remodeling of matrix constituents. The ability to limit the progression of pulmonary emphysema would delay the worst symptoms of the disease and reduce the risk of respiratory failure. However, developing an effective treatment for this disease will require a better understanding of the molecular changes responsible for the distention and rupture of alveolar walls.

A potentially useful approach to studying the relationship between the ECM and alveolar wall injury in pulmonary emphysema involves the concept of emergence, in which interactions at multiple levels of scale produce a phase transition comprising the spontaneous reorganization of a chemical or physical system [11]. The possible role of this phenomenon in pulmonary emphysema emphasizes the need for developing therapeutic agents that concurrently inhibit molecular and higher-order mechanisms of lung injury.

The current paper describes studies that evaluated this therapeutic approach by treating both human and experimentally induced pulmonary emphysema with an aerosolized preparation of hyaluronan (HA), a prominent component of the lung ECM that is significantly reduced in the lungs of patients with this disease [12,13,14,15]. The results indicate that inhaled HA protects elastic fibers from injury, reduces airspace enlargement, and may improve lung mechanics. The potential effects of HA at multiple levels of scale suggest that modification of the lung ECM may provide a model for developing more effective therapeutic agents for pulmonary emphysema.

## 2. Elastic Fiber Injury in Pulmonary Emphysema

### 2.1. The Effect of Elastic Fibers on the Distribution of Lung Mechanical Forces

**Figure 1:**
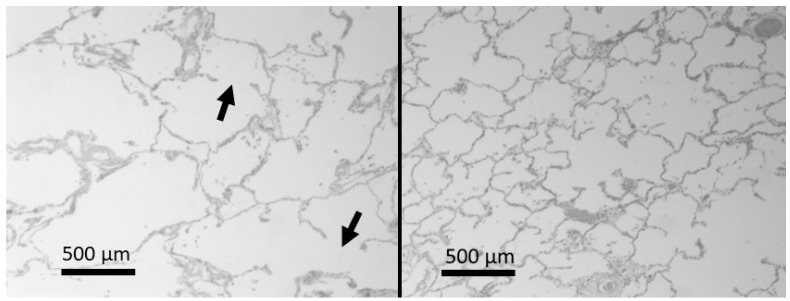
(**Left**) Photomicrograph of a lung with emphysema showing airspace enlargement and alveolar wall rupture (arrows). (**Right**) Photomicrograph of normal lung for comparison. Hematoxylin and eosin. Reprinted with permission [17].

The development of airspace enlargement in pulmonary emphysema involves multiple mechanisms acting on various lung components. However, damage to lung elastic fibers is a significant component of the disease [16,17]. Inflammatory cells recruited by cigarette smoke and other toxins release elastases and oxidants that degrade these fibers, disrupting the forces responsible for air movement through the lung [18,19]. The breakdown of these fibers leads to distention and rupture of alveolar walls (Figure 1), which reduces lung surface area and impairs gas exchange.

Although elastase activity plays a significant role in the development of pulmonary emphysema, there may be other factors that have a more direct impact on the structural changes observed in the disease. Alterations in the transmission of mechanical forces may be necessary to translate the proteolytic damage into the enlargement of air spaces. This idea is supported by in silico studies that demonstrate how local variations in the elasticity of the alveolar walls can lead to widespread architectural changes resembling those seen in pulmonary emphysema [20].

The recoil properties of elastic fibers are based on their ability to store and release energy by changing their shapes. When these fibers stretch during inhalation, they become more organized and decrease entropy. Conversely, they return to a more disordered state during exhalation, providing the force needed to expel air from the lungs [21]. These changes in entropy are influenced by the properties of the elastin core protein, which contains hydrophobic regions that interact with neighboring water molecules [22]. 

**Figure 2:**
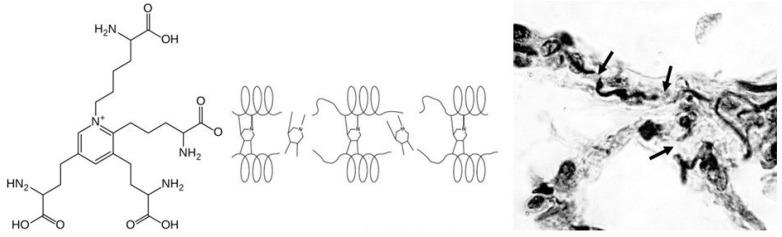
(**Left**) Desmosine is formed by condensation of lysyl residues on adjacent elastin peptides. (**Center**) Loss of elastin cross-links results in unraveling and fragmentation of elastic fibers. (**Right**) Photomicrograph of fragmented elastic fibers (arrows), reprinted with permission [17].

The structural stability of elastic fibers depends on elastin cross-links, including desmosine and isodesmosine (DID), which are formed by the condensation of lysyl residues in adjacent peptide chains and are unique to this protein [23]. Loss of DID due to elastolysis results in the unraveling and fragmentation of these fibers, leading to distention and rupture of alveolar walls (Figure 2). Consequently, the DID level in plasma, urine, and sputum may reflect airspace enlargement.

The importance of these cross-links was demonstrated through the use of the cross-linking inhibitor beta-aminopropionitrile in a model of pulmonary injury induced by cadmium chloride [24]. Treatment with this agent resulted in the development of pulmonary emphysema instead of interstitial fibrosis.

### 2.2. DID as a Biomarker of Elastic Fiber Injury 

As a result of the low turnover of elastic fibers in the absence of lung injury, DID has been used as a biomarker for pulmonary emphysema. These cross-links are elevated in the blood, urine, and sputum of patients with this disease [25,26]. Additionally, higher plasma levels of DID have been associated with decreased lung mass, as determined by high-resolution CT imaging [27]. This finding suggests that monitoring DID levels could provide useful information about the progression of pulmonary emphysema.

**Figure 3:**
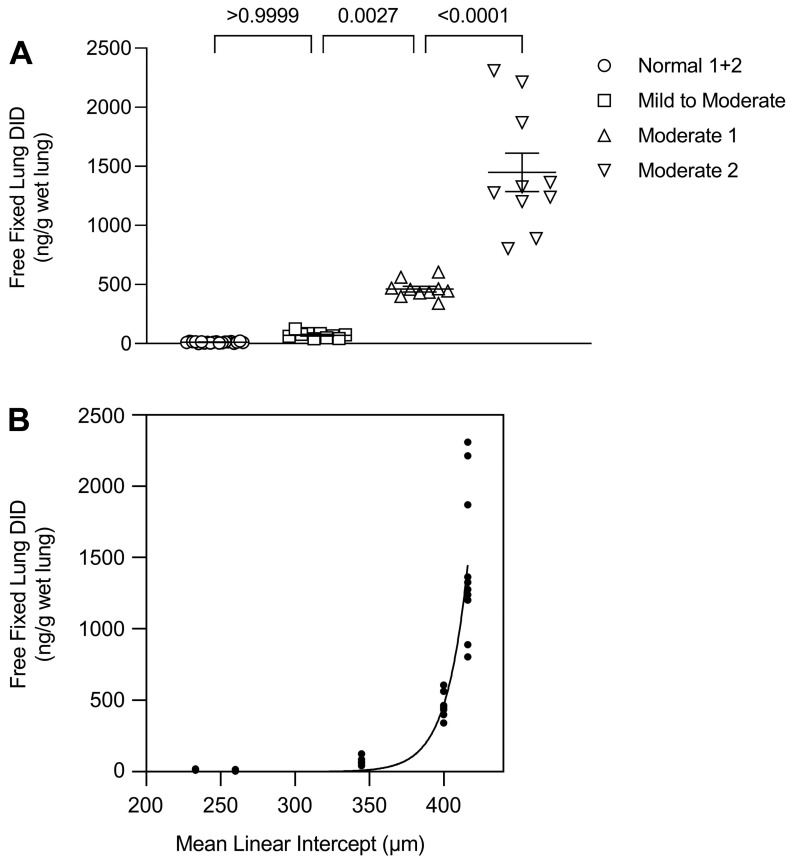
(**A**) Graph showing the release of free DID (not peptide-bound) from normal lungs and those with mild to moderate or moderate emphysema (as indicated by legend symbols). (**B**) Graph showing the relationship between free lung DID and alveolar diameter. A phase transition involving greatly increased loss of elastin cross-links occurs around 400 µm. (**A**,**B**) reprinted with permission [17].

Our laboratory measured the levels of peptide-free DID in hamsters treated with cigarette smoke and lipopolysaccharide (LPS) to induce pulmonary emphysema [28]. The study showed a significant correlation between free lung DID levels and increasing alveolar diameter, suggesting that these cross-links could be a biomarker for airspace enlargement. A similar finding was observed in human postmortem lungs, where the levels of free lung DID markedly increased when the alveolar diameter exceeded 400 µm (Figure 3) [17]. The accelerated release of cross-links from damaged elastic fibers is consistent with a phase transition characterized by the emergence of a clinically apparent disease state.

**Figure 4:**
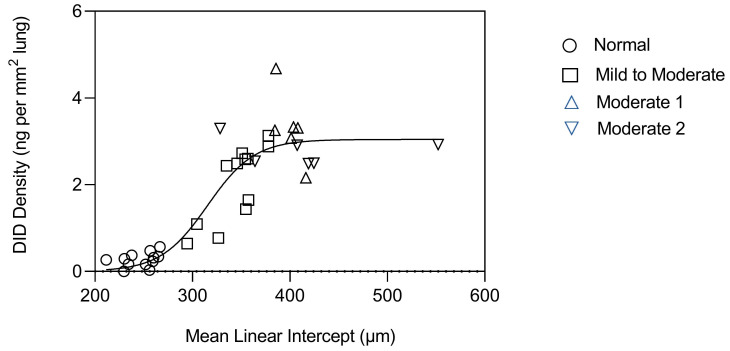
Graph showing the relationship between DID density and alveolar diameter in normal lungs and those with mild to moderate or moderate emphysema (as indicated by legend symbols). A phase transition consisting of a marked increase in cross-link density occurs between 300 and 400 µm. Reprinted with permission [17].

Measurement of DID in tissue sections from these lungs indicated a second component of the phase transition involving a rapid increase in cross-link density at an alveolar diameter above 300 µm, which leveled off around 400 µm (Figure 4). This finding suggests that the early stages of airspace distention are characterized by a balance between injury and repair of elastic fibers, with higher cross-link density associated with increased elastin deposition. Subsequently, the repair of the fibers may progress to a decompensatory stage, where degradation exceeds resynthesis. This resulting loss of structurally intact elastic fibers is accompanied by a phase transition involving accelerated elastin breakdown, alveolar wall rupture, and progression to an active disease state less amenable to therapeutic intervention.

### 2.3. A Percolation Model of Structural Changes in Elastic Fibers

In the early stages of pulmonary emphysema, the destruction of the elastic fiber network in the lungs may be limited to certain areas, causing minimal changes in lung structure. However, as the damage to these fibers worsens, it leads to an uneven distribution of forces within the lungs and the rupture of alveolar walls. This process results in a significant reduction in lung surface area and impairs gas exchange. Percolation theory, which examines the movement of fluid-like substances through interconnected channels, can be employed to model these structural changes [29]. Using this theory, we can explore how small-scale events can have widespread effects on the entire system.

**Figure 5:**
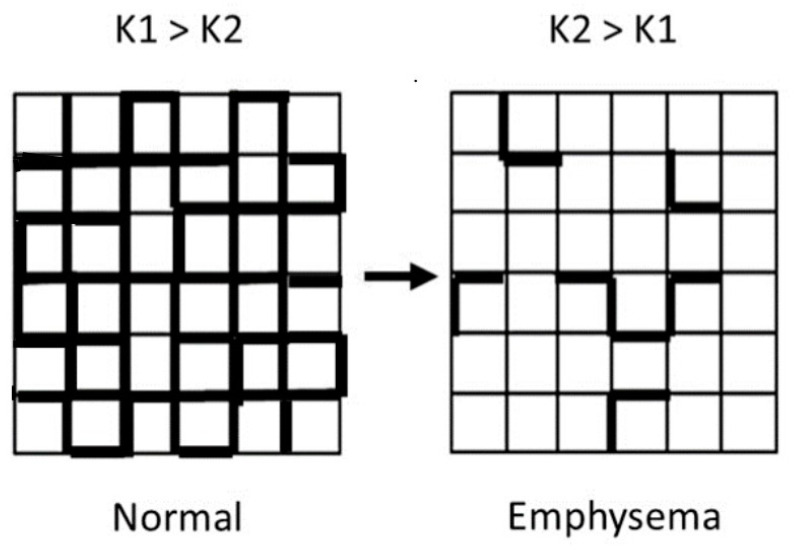
The progression of pulmonary emphysema involves a transition from intact (strong) to fragmented (weak) elastic fibers. The mechanism responsible for this change involves enzymatic and oxidative degradation of the fibers, which increases the mechanical strain on alveolar walls, resulting in their distention and rupture [32].

A percolation model called a random resistor network was utilized to analyze the impact of changes in elastic fibers on lung mechanics [30]. This model randomly disconnects conducting bonds, resulting in increased resistance to flow. The network consists of two interconnected units, labeled K1 and K2, representing intact and fragmented fibers, respectively. These units are randomly distributed within a three-dimensional lattice, and their relative proportions determine how mechanical forces are transmitted through the lung [31]. When there are fewer K2 units, the forces are spread diffusely across the more robust K1 units, minimizing the disruption of lung structure. However, as the proportion of K2 units increases (indicating elastic fiber injury), the forces become increasingly concentrated in the remaining K1 units (Figure 5).

The heightened strain on the K1 units accelerates their breakdown into K2 units, resulting in the loss of lung elastic recoil, hyperinflation of alveoli, and rupture of alveolar walls. These changes are reflected by increased pulmonary compliance and reduced gas exchange, potentially leading to respiratory failure.

### 2.4. The Proinflammatory Activity of Structurally Modified Elastic Fibers

A hamster model of pulmonary emphysema incorporating elastase and LPS was utilized to explore the correlation between lung inflammation and elastic fiber injury [32]. The rationale for combining elastase with LPS was based on studies indicating a synergistic interaction between these two agents with regard to airspace enlargement [33]. In order to strengthen the impact of LPS, a single low dose of elastase was administered to the hamsters, involving a shorter time interval between the instillation of the enzyme and LPS. This adjustment allowed for a more pronounced effect of LPS and facilitated the identification of synergistic interactions between the two agents. Unlike previous studies that included multiple weekly treatments with elastase prior to LPS administration, this modified model focused on a single dose and a condensed timeframe between the two agents [34].

This model was used to determine whether pretreatment with elastase changed the structure of elastic fibers, increasing their susceptibility to secondary injury due to LPS. The results showed that hamsters treated with both elastase and LPS had significantly more BALF cells than those treated with elastase/saline, saline/LPS, or the control group. Moreover, the percentage of neutrophils in the BALF was significantly higher in animals receiving elastase and LPS compared to those receiving elastase or LPS alone.

The proinflammatory activity of elastin peptides released from fragmented elastic fibers was also studied in the LPS model of lung injury [32]. Intratracheal instillation of both elastin peptides and LPS concurrently resulted in significantly increased BALF levels of neutrophils and DID compared to the instillation of either agent alone. The chemoattractant effect of elastin peptides was subsequently determined in vitro using BALF macrophages from untreated animals [32]. While exposure to either elastin peptides or LPS alone significantly increased the level of chemotaxis compared to the control group, the combination of peptides and LPS had a significantly greater effect.

## 3. The Relationship between Collagen and Airspace Enlargement

### 3.1. Collagen Degradation in Alveolar Wall Injury and Repair

Collagen is a significant component of the extracellular matrix in the lungs, providing structural support and maintaining the elasticity of the lung tissue. One of the leading causes of collagen abnormalities in pulmonary emphysema is the increased activity of matrix metalloproteinases [35]. These enzymes degrade collagen fibers, leading to a loss of structural support in the lung tissue. This process is further exacerbated by the reduced expression of collagen-producing cells, such as fibroblasts, in the lungs of patients with emphysema [36].

Alterations in collagen cross-linking have also been implicated in the pathogenesis of pulmonary emphysema. As in the case of elastic fibers, cross-linking plays a critical role in maintaining the stability and strength of collagen. Changes in collagen cross-linking enzymes, such as lysyl oxidase and transglutaminases, can disrupt the integrity of collagen fibers and contribute to the development of emphysematous lesions [37]. The loss of lysyl oxidase activity associated with cigarette smoke exposure can enhance collagen degradation and accelerate airspace enlargement [38]. While the early stages of the disease are characterized by elastic fiber injury and loss of mechanical recoil, the progressive enlargement of airspaces causes a greater strain on collagen fibers, making them more susceptible to degradation. This process may attract additional inflammatory cells, resulting in a self-perpetuating breakdown of collagen [39]. Whether the enhanced turnover of this matrix component results in alveolar wall fibrosis is unclear due to the disparate results obtained from studies of human and experimental pulmonary emphysema [40,41,42].

A more direct determination of the relationship between collagen and airspace enlargement involved the treatment of excised lung tissue with collagenase. Exposure to this enzyme did not change elastic behavior or lung structure, whereas elastase was associated with elastin fiber injury, resulting in decreased mechanical recoil [43]. While these findings suggest that collagen degradation is not directly associated with airspace distention, it may play a role in alveolar wall rupture, where the loss of elastic fibers increases the strain on other matrix components.

### 3.2. Combined Pulmonary Fibrosis and Emphysema

**Figure 6:**
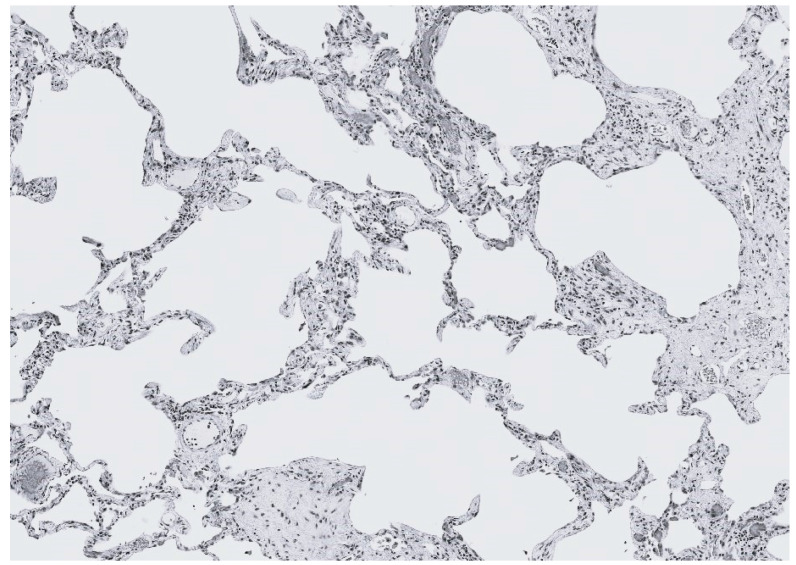
Photomicrograph of a human lung with CPFE, showing a mixed pattern of airspace enlargement and interstitial fibrosis.

The coexistence of distended airspaces and alveolar wall thickening in pulmonary emphysema has led to a new classification called combined pulmonary fibrosis and emphysema (CPFE) (Figure 6) [44,45,46]. The mechanisms contributing to the concurrent presence of different forms of lung damage are still unclear. However, it is believed that cigarette smoke, which can cause both types of lung diseases, plays an essential role in the pathogenesis of this entity [47]. It is hypothesized that exposure to smoke, along with changes in the structure of elastic fibers and subsequent lung injury, triggers an inflammatory response that results in fibrosis of already damaged alveolar walls. This process leads to a complex combination of pulmonary emphysema and interstitial fibrosis.

The juxtaposition of pulmonary emphysema and interstitial fibrosis suggests that they may have a shared underlying cause. It is common to observe fibrotic lesions adjacent to respiratory bronchioles in lungs with centrilobular emphysema. The destruction of elastic fibers, due to the action of elastases and mechanical strain, leads to the recruitment of inflammatory cells. These cells may then release substances that promote scar tissue formation, leading to fibrosis. Therefore, the coexistence of both emphysema and fibrosis may reflect the progression of a related sequence of events rather than the presence of separate disease processes.

Our laboratory investigated the impact of LPS-induced secondary injury on animal models with short-term cigarette smoke exposure or elastase-induced emphysema [32,47]. The findings revealed that the inflammatory processes associated with these models interact synergistically, leading to significantly greater lung disease, as measured by neutrophil content and alveolar diameter. Based on these results, we propose that secondary injury, such as acute exacerbations of chronic obstructive pulmonary disease (COPD), may play a significant role in the development of combined pulmonary fibrosis and emphysema (CPFE).

## 4. The Relationship between Hyaluronan and Elastic Fibers

### 4.1. Hyaluronan Prevents Elastic Fiber Degradation

**Figure 7:**
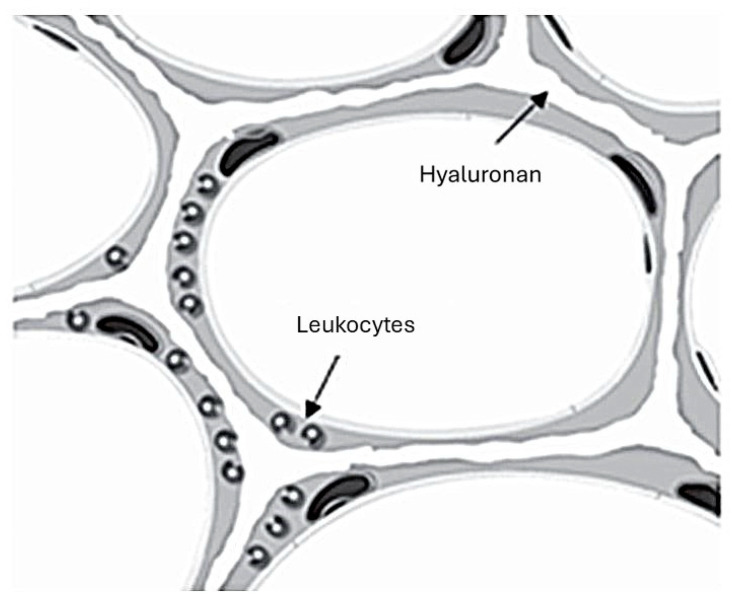
Cartoon illustrating the binding of intratracheally instilled HA to alveolar wall elastic fibers, which protects them from damage due to leukocyte elastases [12].

While most therapeutic approaches to treating pulmonary emphysema have focused on elastase inhibitors, this laboratory has investigated the use of an aerosol consisting of a low-molecular-weight preparation of hyaluronan (HA), a long-chain polysaccharide [12,13,14,48,49]. A previous study showed that pretreatment with hyaluronidase increases airspace enlargement in an emphysema model induced by intratracheal elastase instillation [12]. Conversely, animals pretreated with HA had significantly less airspace enlargement in emphysema models induced by elastase or cigarette smoke [13,49]. This protective effect is due to the binding of HA to elastic fibers, where it functions as a physical barrier against various agents that degrade elastin but not as an elastase inhibitor (Figure 7) [12,48]. The therapeutic potential of supplementing the extracellular matrix with exogenously administered HA is further supported by a study showing a significant decrease in lung HA levels in patients with alpha-1 antiprotease deficiency-induced pulmonary emphysema [15].

To determine whether HA was effective against other types of elastase, we used cell-free radiolabeled elastic fiber-rich matrices, which were coated with HA and exposed to either human neutrophil elastase or human MMP-12 [49]. The results indicate that HA significantly decreased the elastolysis induced by both enzymes, indicating its potential usefulness in treating human emphysema caused by various injurious agents.

The attachment of HA to elastic fibers may involve the formation of electrostatic or hydrogen bonds. Such binding sites may not be situated on the elastin protein itself but may instead involve the surrounding microfibrillar component of elastic fibers [50]. Due to its self-aggregating properties, the inhaled HA may produce large complexes that more effectively protect elastic fibers against elastases and the cells that secrete them [51].

The clinical efficacy of HA was evaluated in a 28-day trial using free DID as a biomarker to assess the efficacy of HA in patients with pulmonary emphysema induced by alpha-1 antiprotease deficiency [14]. Inhalation of aerosolized HA twice daily resulted in a significant decrease in free DID levels in urine over the course of the trial. In other studies, HA was also shown to attenuate the decline in pulmonary function in patients with acute exacerbations of chronic obstructive pulmonary disease (COPD) and prevent bronchoconstriction in patients with exercise-induced asthma [52,53].

Since elastic fiber breakdown may be a final common pathway in pulmonary emphysema, HA might be effective against multiple agents capable of causing alveolar wall injury. The slow progression of emphysematous changes suggests that even a small decrease in airspace enlargement could significantly impact the disease. By protecting elastic fibers from enzymatic and oxidative damage, aerosolized HA could alter the natural history of pulmonary emphysema, thereby reducing the risk of respiratory failure.

### 4.2. Effect of HA on the Mechanical Properties of Elastin

In addition to preventing elastic fiber injury, HA may prevent airspace enlargement by improving the mechanical properties of elastin. The negatively charged carboxyl groups within HA repel each other in solution, expanding its domain and allowing it to trap water [54]. This property is crucial for regulating lung hydration, as the loss of HA from the lung interstitium has been shown to reduce extravascular water content [55]. The interaction between water molecules and hydrophobic amino acid groups in elastin is responsible for elastic recoil [22]. When elastin is distended, water molecules surround its hydrophobic domains, resulting in a large positive change in free energy that provides the force necessary to remove air from the lung and prevent alveolar wall distention and rupture.

The mechanical properties of elastic fibers may also depend on the intramolecular forces of attraction among the elastin peptide chains [54]. The loss of water reduces the distance between the elastin peptide chains, increasing their cohesion and reducing the distensibility of the fibers. At the extreme, the complete absence of water may cause the fibers to become brittle, making them susceptible to rupture under strain. The resulting breakage of the fibers could mimic the effects of enzymatic degradation.

Studies of human lung tissue from patients with pulmonary emphysema have demonstrated a significant reduction in HA content, which would adversely affect extravascular water content [15]. It is anticipated that the administration of exogenous HA will increase the density of water around the dehydrated elastic fibers and improve their mechanical properties. This process might be enhanced by the self-aggregation of HA, which could further entrap water in the proximity of elastic fibers.

While it remains to be seen what effect exogenously administered HA will have on pulmonary mechanics, indirect evidence exists that it may increase lung water content. Rats exposed to nebulized HA for 14 days showed a dose-dependent increase in lung weight not due to cellular proliferation (unpublished data). Microscopically, there was no evidence of pulmonary edema, suggesting that any increase in lung water was bound up in the extracellular matrix. Furthermore, the clinical trial involving a 28-day treatment with HA did not indicate any adverse reactions involving pulmonary edema [14].

## 5. Therapeutic Considerations

A more sensitive measure of therapeutic efficacy would expedite the development of therapeutic agents for pulmonary emphysema. Clinical trial endpoints for this condition are currently limited to pulmonary function tests, which can take a long time to show measurable results [56]. An alternative endpoint is high-resolution computerized tomography, which is more sensitive but still requires a significant amount of time to detect positive outcomes [27].

As shown in the clinical trial of HA, free DID in urine may be a biomarker for the progression of pulmonary emphysema [14]. Despite the potential influence of co-existing diseases involving elastic fiber damage, such as atherosclerosis or osteoarthritis, on the specificity of free DID as a biomarker, it could nevertheless play a critical role in clinical trials. Significant differences in cross-link levels between closely matched experimental and control groups would demonstrate therapeutic effectiveness.

Furthermore, measuring free DID in sputum and breath condensate could enhance the specificity for pulmonary emphysema. However, for free DID to be accepted as a biomarker for this condition, it will be necessary to develop an accurate and reproducible analytic method. Currently, there is no standardized protocol for measuring free DID, and the cost of expertise and equipment may pose challenges to the widespread adoption of this biomarker.

Regarding the treatment of pulmonary emphysema, potential agents that target multiple mechanisms involved in the pathogenesis of the disease may have increased efficacy. Current treatments that focus on a specific component of the inflammatory process, such as elastase inhibitors, have shown limited effectiveness in treating the disease. This finding may be due to the complex nature of the disease, where various interactions at different levels of scale contribute to its progression rather than the activity of individual molecules. As a result, even if specific molecular factors are addressed, the overall structural changes in the alveolar walls may still impact the progression of the disease.

## 6. Future Directions

Pulmonary emphysema is a lung disease characterized by changes in the structure of the lungs, such as enlarged airspaces and fibrosis. Detecting the disease at an early stage requires understanding the molecular and larger-scale mechanisms that indicate the development of the disease. In silico modeling can help identify changes in cellular events before the disease becomes clinically evident. Having a sensitive biomarker for pulmonary would enable early intervention with appropriate treatments.

Regarding treatment, the complexity of pulmonary emphysema suggests that a single drug may not be capable of ameliorating both the emphysematous and fibrotic components of the disease. Consequently, future clinical trials designed to treat pulmonary emphysema should consider using agents with multiple therapeutic effects, such as aerosolized HA. This concept is supported by a recent study showing that HA mitigates COPD exacerbations, a condition involving mechanisms of lung injury that could circumvent the activity of more specific anti-inflammatory agents.

An alternative to treating pulmonary emphysema with a single agent could involve the combination of elastase inhibitors with drugs that have a different mechanism of action. One or more inhibitors could be delivered in a vehicle containing HA or another agent that might potentiate the anti-elastase activity of the other components. This approach to treatment could include combining elastase inhibitors with drugs that decrease inflammation or induce elastic fiber resynthesis.

## 7. Conclusions

Pulmonary emphysema involves multiple pathogenetic mechanisms, including excess elastase activity, unevenly distributed mechanical forces, and oxidative stress. The interaction of these various processes may lead to converging patterns of injury, including loss of collagen and elastin cross-links, microscopic fragmentation of elastic fibers, and macroscopic rupture of alveolar walls. The progression of the disease at different levels of scale provides a rationale for developing drugs that inhibit the broader process of disease emergence rather than individual components of the inflammatory reaction associated with airspace enlargement. Treatment with exogenous HA may accomplish this objective by protecting elastic fibers from various insults and preserving the mechanical properties of elastin. However, the potential efficacy of HA and other therapeutic agents may depend on the early detection of alveolar wall damage before there is extensive airspace enlargement. While there is no accepted method for accurately measuring the initial stages of pulmonary emphysema, using free DID as a biomarker for elastic fiber injury size may permit timely therapeutic intervention that reduces the risk of respiratory failure.

## Data Availability

Not applicable.
